# Multiplying Licensing Boards

**Published:** 1905-02

**Authors:** 


### TEXAS MEDICAL JOURNAL.

AUSTIN, TEXAS.

A MONTHLY JOURNAL OF MEDICINE AND SURGERY.

EDITED AND PUBLISHED BY
F. E. DANIEL, M. D.
Mrs. F. E. DANIEL, Business Manager.

Published Monthly at Austin, Texas. Subscription price $1.00 a year in advance.

Eastern Representative: John Guy Monihan, St. Paul Building, 220 Broadway New York City.

Official organ of the West Texas Medical Association, the Houston District Medical Association, the Austin District Medical Society, the Brazos Valley Medical Association, the Galveston County Medical Society, and several others.

### MULTIPLYING LICENSING BOARDS.

The legislators are the trusted guardians of the people's rights and interests. There are none more sacred than those of life and health. They doubtless are sincere in the desire to safeguard these, as all other interests are safeguarded, but they do not realize, as do the educated physicians (without whose counsel no intelligent protective legislation is possible) the magnitude of the danger of an uneducated "doctor." He is a factor potent for harm, and to turn him loose upon a community, as the Texas law now does, is little short of criminal.

That the State should have a reasonable standard of educational requirements to which all should conform who are allowed to practice medicine (with or without drugs) needs no argument or defence. As there is but one Christian religion, but numerous "sects" or "denominations," so there is but one science of medicine (one medical profession), but many phases of practice, as exemplified in the numerous "pathies." Fundamentally they are the same; all schools teach, or are supposed to teach, the elementary branches (anatomy and physiology being the basis of medical education, whatever the methods of practice may be), and a knowledge of them is indispensably necessary. It was a mistake to ever have made more than one board of examiners, one board should have examined applicants of different schools only on the branches that are common to all. Now, already we have three, and to create a separate board for each sect, would be confusion worse confounded, and a reproach to the State; a board whereby a set of unknowns shall be licensed to practice by a half dozen equally unknown, of their own choosing. Why should the physio-medicals, whatever they may be, and the Osteopaths (whose very name is an absurdity: it means "diseased bones") not be examined by one of the existing boards? The efforts to secure a separate board for their especial benefit, when they are an infinitesimal part (less than 2 per cent) of the profession, is a dodge to escape examination at the hands of the State's reperesentatives, the existing boards. Naturally these parties squeal and shuffle when the State requires of them a degree of knowledge they know they do not possess. If they are educated in the common branches of medicine, as they claim to be, why are they afraid to be examined by the trusted representatives of the State—men of known integrity, honor and learning? Should the Legislature commit the unwise act of creating an examining board for one "pathy," a hundred strong, there would be nowhere to draw the line, and every other sect would demand the same. Texas would become the trash pile for all other States and would be overrun by the quacks and incompetents driven out by wise and proper laws. The State would become an object of ridicule and pity. The people must be protected. To the majority of them, a "doctor" is a "doctor." They can not discriminate between a physician and a fake. What a figure Texas would cut with her State Board of Medical Examiners; her Homœopathic Board; her Eclectic Board; her Osteopath Board; her Physio-Medical, Vitopath, Magnetic Healers, Faith Healers; her Christian Science Board. Add, then, a board for the voodoo doctors, and the reductio ad absurdum will be complete. "Let slip the dogs of war and cry 'havoc.'" And may God have mercy upon the innocent and ignorant public.

The bill creating a Board of Osteopath Examiners passed the Senate February 7th by a majority of one. Seven Senators were absent, amongst them Senator Davidson who last session so ably championed the cause of legitimate medicine and protection of the public against quackery. Senator Hicks voted for the bill. Senator Decker took up the cudgel for the protection of the public from uneducated "doctors" and resorted to the usual tactics of delaying action, by a call of the Senate; but next morning the absentees were excused and on a showdown the bill passed, 13 to 11, as follows:

Yeas—Beaty, Griggs, Hale, Hanger, Hawkins, Hicks, Hill, McKamy, Skinner, Smith, Stafford, Stone, Willacy.

Nays—Barrett, Brachfield, Chambers, Decker, Faust, Grinnan, Harper, Looney, Martin, Stokes, Terrell.

Absent—Davidson, Harbison, Holland.

Absent-Excused—Faulk, Glasscock, Meachum, Paulus.

Paired—Senator Holland, present, who would vote nay, with Senator Faulk, absent, who would vote yea.

Senator Grinnan made a strong presentation of the question from a rational standpoint, and vigorously opposed the passage of the bill. So did Senator Looney. The physicians of Texas will not soon forget them. Mr. Grinnan showed that at last session of the Legislature these parties "claimed exemption from the law because, they said, they are not a medical school and do not practice medicine. *Now* they claim they *are* a medical school and (the courts having so decided) that they are practicing medicine, and are learned in all the branches of medicine common to all schools, yet ask to be excused from examination by the existing boards, and want to examine each other. Why? Because they know they can not stand the required examination, and this demand for a separate board is a dodge to escape what is required of others."

There is a handsome young Senator of whom it is reported that he took "absent treatment" from the Witmers or Wellmers—whoever they are—some two years. He voted for the bill. He preferred the "allopaths," he said, but his neighbor ought to have the right to send for an "osteopath" if he desired. That is the quintessence of rot. No one on earth objects to anyone employing an "Os," or a "phys," or any other pathy, but, in the name of reason, require that "Os" or "phys" or voodoo to satisfy the State that he is educated sufficiently to be entrusted with the exercise of a privilege dangerous to the public.

The State Medical Association earnestly advises the enactment of protective legislation. It seems impossible to make the average legislator see the matter as we see it. They can not be made to understand that the practice of medicine does not consist of giving drugs. That is the smallest part of medical science. It is more important and easier to prevent than cure disease. "Medicine" is the healing art, and embraces prevention. The means and methods of healing—material or immaterial—are "medicines." Thus a drug is a medicine, but all medicine is not drugs. A medicine is a curative agent, whether that agent be drug, faith, emotion, electricity, massage or manipulation or prayer. Else why do we always say "drugs *and* medicines" if they are one and the same thing?

But what's the use? When we see a sect of less than one hundred unknown persons able to neutralize the influence and defeat the advice and wishes of the combined medical profession of the State, it is clearly useless to argue the question.

A doctor—friend of mine—was asked to see Sarah. He examined her and was leaving, without a word, when the mother said: "Doctor, what's the matter with my daughter?"

"What do *you* think is the matter with her?" asked the doctor.

"I'm afeered she has got the nine months sickness," said mama.

"That's what's the matter," said the doctor. The mother wept, and wringing her hands, sobbed out: "I've *talked* and *talked* to Sarah." "Yes," said the doctor, "but some man has out talked you." They have out talked us (so far).

The next in order is a bill for a separate board for the physio-medicals, which is following close upon the heels of the osteo bill, and there is a very general belief that it will pass. Next! Bring on your Christian Science board and your voodoo board. I wish to suggest that no voodoo be permitted to practice unless he can read and write, and that Dr. Sam be president of the board. Respectfully submitted.

---

February 22d.—The publication of this number of the Journal having been delayed, by a press of State printing, far beyond its date of issue, I am enabled to give later news of the status of medical legislation: The Osteopath bill (Senate Bill No. 61) (Hanger's) went to the House, and together with the House Bill (Cottrell's) was referred to House Committee on Public Health. They reported adversely upon them, and a subcommittee of this committee was appointed to present a substitute bill. To date it has not been reported.

Nothing has been done with the Anatomical Bill, further than its introduction in the Senate by Looney and in the House by Witherspoon.

The State Medical Association's bill amending the Medical Practice Act was introduced in the Senate by Davidson on the 15th, referred to Judiciary Committee No. 2, of which Senator Davidson is chairman. Senator Davidson had been chosen by our Committee on Public Health and Legislation to introduce and advocate the bill, but he was almost continuously absent till about the 15th, hence the delay.

Drs. Daniel, M. M. Smith and J. S. Wooten, by invitation, appeared before the committee on the 18th and again on the 21st. They presented the case on behalf of the State Medical Association, stating that this bill has the endorsement of the entire State Association and is authorized by its House of Delegates, and that it is believed will do much to protect the public from ignorant persons who essay to practice of medicine. Dr. Daniel said it is by no means the wish or intention to persecute, oppress or "cut out" or exclude the competent of any "school" or sect, or quarrel with those who differ with us on modes of practice. It is the intention of the bill to exclude the incompetent and unqualified of every school or sect. The bill is a transcript of the existing law—three boards of nine each—amended so as to remedy the great defect; the clause that exempts from all restrictions a class of persons solely because they do not give drugs; and to require them to be examined in all branches of medicine, except materia medica and practice. It is eminently fair. One representative of the Osteopaths who was present said, when questioned by the chairman, that his people are willing to be examined in the branches named, if they could have a fair examination—which goes without saying. Pushed, he acknowledges that what they are fighting for is recognition,—and in reply to a question from Senator Looney, he said there are "about 125 *regular* Osteopaths in Texas, recognized, and about 225 more—350 in all—who were pretenders, and that they should be examined in the principles and practice of Osteopathy!" And the 125 want a bill to examine them.

The fight against our bill will be made by the Christian Scientists. I think they should be exempted from examination in medicine, because they do not pretend to medical knowledge. To require them to be examined will be equivalent to prohibition, and sentiment is against their total suppression, and sympathy with them. If they are prohibited by law under penalty from undertaking any acute disease or surgery or obstetrics, and compelled to call a licensed physician to those cases, they can do no harm. I think this is the solution of the problem, and I advise it.

LATER: February 22d. Judiciary No. 2 turned us down, and appointed Looney and McKamy to report a substitute bill. It will be a mixed board, giving every pathy a member except Christian Science, the X-ray eye man, and Dr. Sam. Davidson refused to let the amendment bill be withdrawn.

THE PATENT MEDICINE BILL, as it is called, introduced in the House last week by Witherspoon, "by request" of whom, I do not know, strikes at the secret nostrums that are recognized by the medical profession as a great menace to the public health and safety; the nostrums advertised in the newspapers, illustrated with United States Senators and Generals and Lords and Ladies galore, who testify to the wonderful virtues of the stuff. It is an evil that should be regulated by a State Board of Health—if we had one. Many of these contain alcohol, opium and cocaine, and, doubtless, to cheapen the production wood alcohol has been substituted for alcohol. Wood alcohol in repeated doses causes blindness; in large doses—death.

But unfortunately the bill makes no discrimination between these nostrums and the legitimate pharmaceuticals known as proprietary medicines—trade marked—not "patented"—by the United State government. These, many of them, are manufactured for,—advertised solely to,—and extensively prescribed by physicians. It is pharmacy on a large scale, and they are rational preparations, indispensable to the physician and are staple articles in many households.

If this bill should become a law it will ruin the retail drug trade—entail much inconvenience to the medical profession, who so largely prescribes them, and privation and hardship on hundreds of families. It will drive the proprietary medicines out of Texas entirely, because if the manufacturers are compelled to publish their formula (any physician can get it, and most of them know the formula of the principal articles), the substituting druggist will imitate it. The bill should be amended so as to discriminate between such preparations as "Peruna" and "Swamp Root" and "S. S. S," on the one hand and Listerine, Tongaline, Bromidia, Fellows' Hypophosphites, Cascara (Kasagra), etc., on the other hand. Under the provisions of the bill, a person can not, without a prescription, buy a bottle of liniment or cough syrup or a box of C. C. pills.

I hope my readers will at once write to their representatives, asking them to modify or defeat the bill. It should apply only to the villainous nostrums advertised directly to the people in the newspaper.

---

A STATE SANITARIUM FOR CONSUMPTIVES.—As I go to press, I learn that Mr. Wilmeth has introduced a bill for a sanitarium for consumptives. I gave him some data, but have not seen the bill.

